# The complexity of reaching and maintaining a healthy body weight – the experience from adults with a mobility disability

**DOI:** 10.1186/s40608-018-0212-6

**Published:** 2018-12-03

**Authors:** Marianne Holmgren, Magnus Sandberg, Gerd Ahlström

**Affiliations:** Department of Health Sciences, Lund University, P.O. Box 157, 221 00 Lund, SE Sweden

**Keywords:** Mobility disability, Overweight, Qualitative research, Prevention

## Abstract

**Background:**

People with a disability affecting their mobility are more likely to be overweight or obese than those without a mobility disability. The guidelines on how to prevent and treat overweight/obese adults in the general population have not been adapted to the needs of people with a mobility disability. A reasonable useful first step in the process of adapting such guidelines is to conduct a qualitative study of the perceived needs of these people.

**Aim:**

The aim was to explore the experienced importance of body weight among adults with a mobility disability and their perceived needs and actions to reach and maintain a healthy weight.

**Method:**

This was an explorative qualitative study based on individual interviews and qualitative content analysis. An inductive analysis of the interviews formed the basis for the establishment of sub-categories, main categories and, finally, a main theme. The twenty participants included in the study have had a mobility disability for more than two years before being recruited.

**Results:**

The overall theme, “The complex trajectory to a healthy weight”, included four main categories. In the category (i) *Vicious circle of problems*, the participants perceived that everything was harder with the combination of a mobility disability and being overweight/obese with one factor making the other worse. In (ii) *Strategies based on decisions and attempts*, the participants talked about different ways of attempting to reach or maintain a healthy weight. In (iii) *Internal resources*, they spoke of awareness and motivation as contributory factors. In (iv) *External resources — experienced and required*, they spoke about feelings that their weight problems were not given high priority in primary health care. They found it difficult to get advice designed for persons with a mobility disability and felt that competence was lacking among health professionals. The participants asked for a team of professionals with adequate knowledge concerning mobility disabilities.

**Conclusions:**

People with a mobility disability combined with being overweight/obese have a complex living situation and health needs. The experiences communicated by participants may facilitate adaption of existing intervention programs or development of a new evidence-based obesity prevention program for primary health care settings.

## Background

Being overweight and obese are more common among people with a mobility disability (MD) than among the general population [1–4]. Being overweight, with a prevalence of 39%, and obese, with a prevalence of 13%, are a global health concern in the adult population worldwide [5]. There are similar trends in Sweden [6]. In addition, being overweight/obese increases the risk of many chronic diseases, such as diabetes, stroke and certain forms of cancer that all contribute to higher risks of mortality [5, 7]. While evidence-based guidelines to prevent and treat being overweight/obese in adults exist [8, 9], we are not aware of such guidelines adapted for people with an MD [10].

About 16% of the adult population worldwide has some kind of disability [11]. For Sweden, the corresponding figure is 21%, and 7% live with an MD [12]. People with an MD are at greater risk of developing chronic diseases, such as diabetes and high blood pressure [13, 14], as well as other conditions, such as obesity, depression, social isolation, pain and fatigue [4, 15, 16]. In treatment and prevention of obesity-related diseases, regular physical activity is a key determinant of energy expenditure and fundamental for energy balance. At the same time, it reduces the risk of heart disease and other diseases [17, 18]. However, an MD often makes it difficult to sustain regular physical activity [19, 20]. Therefore, healthy eating habits may be the primary target for people with an MD to control their weight. Contributing reasons for unhealthy weight (overweight and obesity) among people with disabilities may be the use of food as a consolation for sadness and boredom or as a consequence of loneliness or the strains of everyday life [3]. Unhealthy weight in people with a disability may also be due to too high intake of energy dense food that is typically cheaper and easier to prepare [21]. To support individuals with an MD who are overweight/obese, improving their eating patterns may be more challenging compared to individuals without an MD. Therefore, it seems important to explore resources, motivation and potential strategies for reaching or maintaining a healthy weight (normal weight) among individuals with this combined disability.

Despite the literature on barriers to and facilitators of healthy eating habits [22–24] and physical activity [25–27] among people with an MD, research on adapted intervention programmes is still sparse. Such programmes constitute one way of promoting physical activity and healthy eating habits among people with an MD. By involving people with an MD [28], evidence-based health promotion programmes designed and evaluated for the general population [10] may be further tailored to the needs of people with an MD.

Even though qualitative research is sparse in this area, the existing studies revealed complexity. In a qualitative study by Nosek and colleagues on the meaning of health, [29] women with an MD referred to a complex web of psychological, social and environmental factors that influence well-being and generate difficulties for maintaining good perceived health and a normal weight. A qualitative study by Mudge et al. [30] revealed that the views of people with an MD for quality of life go beyond the prevention of obesity. Instead, the views emphasised social and emotional well-being. The participants described complex social and emotional processes influencing their possible decisions and ability to prioritize improvements in healthy eating and physical activity. However, the study by Nosek et al. included only women, and the study by Mudge et al. included people with a wide range of disabilities. To be able to help people with an MD maintain a healthy weight or lose weight, research on the weight-related struggles of these people is needed. Such research is crucial for the development of health-promotion programmes specifically designed for people with an MD or the adaptation of programmes designed for the general population.

## Aim

The aim was to explore the experienced importance of body weight among adults with a mobility disability and their possible paths to reaching or maintaining a healthy weight.

## Method

### Design

The current study has an exploratory qualitative design and is based on individual interviews with adults with an MD. Ethical approval was obtained from the Regional Ethical Review Board in Lund (Dnr 2015/773).

### Sample

The participants were recruited from the Skåne Public Health Survey (SPHS) conducted in 2012. Eligible people participated in the SPHS and indicated an interest in receiving further information about future studies (*n* = 403). The inclusion criterion for an MD was that during two years prior to participating in the SPHS, the person was unable to run 100 m and was unable either to take a single step on a stair or to take a five-minute walk. A purposeful selection was made with the aim of achieving variations in age (18–64 years), sex and weight (underweight/normal weight, overweight and obese). The following weight classes were included according to body mass index (BMI): underweight < 18.5 kg/m^2^, normal weight ≥ 18.5 < 25 kg/m^2^, overweight ≥25.0 < 30 kg/m^2^ and obesity ≥30 kg/m^2^ [31]. The first author (MH) sent an informative letter about the study to individuals who were eligible to participate and contacted them by telephone a week later to ask whether they had any questions about the study and whether they were willing to participate. A total of 26 people were approached and invited to join the study, and 6 declined participation because of a lack of energy or lack of interest. Ultimately, a total of 20 participants were included in the study.

The age range of the study group was 36–67 years, and most of the participants were married and took early retirement because of their disability. Several participants experienced tiredness, pain, sleeping problems because of pain and a limited social life (Table 1).

**Table 1:** Background characteristics and health status

Fictitious name	Age	Marital status	Employment status	BMI	Pain	Tiredness/ Fatigue	Help with personal care	Trouble with sleep	Influence on social life	Travel
Michael	40s	Married	Studying	24.2	No	No	Yes	No	No	Yes
Johnny	50s	Married	Retired	32.2	Yes	Yes	Yes	Yes	Yes	Yes, short trips
Jonna	30s	Married	Unemployed	41.3	Yes	Yes	No	No	No	Yes
Maria	50s	Partner	Working	33.5*	Yes	Yes	Yes	Yes	Yes	Yes
Klas	60s	Married	Working	31.9	Yes	No	No	Yes	No	Yes
Ingemar	60s	Married	Retired	32.1	Yes	Yes	No	Yes	No	Yes, short trips
Kristina	50s	Single	Retired	21.0	Yes	Yes	No	Yes	Yes	Yes
Niklas	50s	Divorced	Retired	27.2	Yes	Yes	No	Yes	No	Yes
Erik	60s	Live-apart	Retired	22.7	No	Yes	No	Yes	Yes	Yes
Filippa	40s	Single	Unemployed	26.2	Yes	Yes	No	Yes	Yes	Yes
Elisabeth	50s	Married	Retired	29.0	Yes	Yes	Yes	Yes	Yes	Yes, short trips
Gunilla	50s	Married	Unemployed	33.8	Yes	No	No	No	No	Yes
Karl	50s	Divorced	Retired	27.7	Yes	No	No	Yes	No	Yes
Christoffer	40s	Single	Retired	29.9	Yes	Yes	No	Yes	No	Yes
Britt	60s	Married	Retired	30.9	Yes	Yes	Yes	Yes	Yes	Yes, short trips
Therese	30s	Married	Work	25.0	Yes	Yes	Yes	Yes	No	Yes
Caroline	50s	Single	Unemployed	25.9	Yes	Yes	No	Yes	Yes	Yes, short trips
Eva	60s	Married	Working	24.6	Yes	Yes	No	Yes	Yes	Yes
Margareta	60s	Live-apart	Retired	23.7*	Yes	Yes	Yes	Yes	No	Yes, short trips
Peter	50s	Single	Retired	31.6	Yes	Yes	Yes	No	Yes	No

* Missing data, BMI from SPHS 2012

### Individual interviews

Interviews with all 20 participants were conducted between April and September 2016 by the first author (MH). The participants chose the time and place for their respective interviews. Before each interview started, MH repeated the information about the aim of study and reaffirmed that participation was voluntary and included the right to cancel the interview at any time. Written informed consent was obtained before the interview started, and the participants were asked to answer a short questionnaire about sociodemographic characteristics (Table 1). The interviews were digitally recorded and then transcribed verbatim. They lasted between 27 and 99 min.

The interviews were semi-structured in that an interview guide developed specifically for the study was used, and it was designed to capture the participants´ experience of weight-related problems. The interviews were conducted as a dialogue [32]. The interview guide included the following four broad open-ended questions and a number of follow-up questions:

1. Could you say something about how you feel about your weight in relation to your mobility disability? What weight do you think would be ideal for you?
2. In what ways do you think your daily life would be different if your weight was different?
3. How have you taken care of your weight the last two years? Have you found any special ways to do it?
4. When it comes to getting to, or remaining at, the right weight, what support have you received from your family? What support have you received from other people in general and from primary health care staff?
  The number and formulation of follow-up questions depended on the richness of the participant’s answers to the main questions. The follow-up questions were typically designed to draw out information that is more specific: “In what way...?”; “Can you give me an example of...?”; “What kind of support...?”

### Analysis

The interviews were subjected to a qualitative content analysis in terms of a division into sub-categories and categories as described by Graneheim and Lundman [33]. Initially, the first author (MH) read each interview several times to get a sense of the overall interview. Next, the meaning units were identified, then condensed and labelled with a code. These codes were used to generate sub-categories based on differences and similarities at a manifest level. The sub-categories were then sorted into main categories. Finally, from the main categories and sub-categories, a main theme emerged for interpreting all of the content. In the analysis, the 10th version of the NVIVO software [34] was used as a tool for sorting the text and establishing different levels of abstraction. The co-authors (MS and GA) read several of the interviews, all the meaning units and the sub-categories independently to acquire an understanding of the data. Next, they discussed the codes, the main categories and the theme with MH. Finally, all authors discussed the categorization until a consensus about the final results was reached. Examples of the analytical process are presented in Table 2.

**Table 2:** Examples of the analytical process

*Meaning units*	*Condensed meaning units*	*Code*	*Sub-category*	*Main category*	*Theme*
Lot of things give overweight: medication, food, disability, bad sleep and stress. The list is endless, and you always have a bit extra of everything when you’re living with chronic pain or chronic mobility disability. There it is: if you’re sitting in a wheelchair you’re sitting in a wheelchair. And if, like me, you’ve got chronic pain, it’s so much worse. It makes such a — *such a* — difference.	Overweight, pain and poor sleep in relation to mobility disability	Several problems	Problems	Vicious circle of problems	
It depends on how you are. But it’s difficult for me to think of putting all my energy into losing weight —hardly eating anything at all, crash-dieting. I suppose my method’s been to eat just enough and reduce weight over a long period.	Long-term perspectives is necessary for weight reduction	Thinking long- term	Strategies tried	Strategies based on decisions and attempts	The complex trajectory to a healthy weight
So I’ve followed that kind of guidelines pretty much—but as I said, when you think things are at their best and you stop following the guidelines and sit yourself down on the couch and think “Now I’m at my peak”, you don’t stay that way very long, because everything’s perishable.	One cannot believe that it can work by itself	Insights	Awareness	Internal resources	
I’d thought it’d be really good to have someone to talk to about it —some kind of (how shall I put it?) coach or something... Someone who understood what it’s like to have fibrositis.	Asking for tailored advice	Advice	Required support	External resources — experienced and required	

## Results

The participants’ experiences of the significance of body weight in everyday life and their path to reach and maintain a healthy weight were captured in the theme: **The complex trajectory to a healthy weight**. They saw all challenges and problems as a complex inter-connected webb. Irrespective of the specific nature of their own difficulties, of their own attempts to manage or of their own need for support, the problem they were confronted with was complex, and the participants needed to be properly understood. The theme consists of four main categories: *Vicious circle of problems*, *Strategies based on decisions and attempts*, *Internal resources* and *External resources — experienced and required*. Each category includes 3–7 sub-categories (Table 3).

**Table 3:** Overview of main categories and sub-categories

Theme	The complex trajectory to a healthy weight			
Main categories	*Vicious circle of problems*	*Strategies based on decisions and attempts*	*Internal resources*	*External resources —experienced and required*
Sub-categories	Mobility disability	Tried to do	Awareness	Informal support
	Overweight and obesity	Decided to do	Motivation	Formal support
	Pain	Regular mealtimes	Attitudes	Required support
	Impaired mental well-being	Believed in special diets	General thinking	
	Difficulty in exercising	No faith in special diets		
	Lack of sleep and fatigue	Long-term perspectives		
	Limited outdoor exercise	Physical activity or exercise		

### Vicious circle of problems

Participants spoke of being in a vicious circle. Problems that originated with their *mobility disability* were exacerbated by the problems of being *overweight* and *obese*, which exacerbated the other problems in turn. Everything got harder with the combination of having an MD and being overweight or obese. Many health problems were connected to each other and one led to another. Being overweight or obese exacerbated the MD and the *pain*, which affected the ability of the participants to exercise. They also felt that being overweight or obese led to *impaired mental well-being* and to a tendency to use food as a consolation for sadness, which in turn led to additional weight gain, worsened the MD and led to additional pain. In addition, being overweight or obese affected participants who said “your belly gets in the way”, which made it difficult to bend down to put on socks and shoes, for example. One man described a complex situation in the following way:

*With titanium in my back and a belly that gets in the way, I was hampered a lot. Then there’s the tiredness.... It’s not just because of the weight itself, although your weight does make it hard for you to move and hard for you to keep fit. Pain in the hip is another issue that it makes it difficult for you to move. So, yes, I feel I can say that my weight was quite an obstacle.* (Christoffer)

The participants spoke of how an MD and pain often went hand in hand. They felt that it was not only the MD that limited their mobility but also their pain, which caused *difficulty in exercising* and restricted their daily activities as well as *lack of sleep and fatigue*. The resulting difficulty in exercising contributed to being overweight and obese. Exercise can exacerbate both the disability and the pain. As one of the participants put it, “there’s a price to pay”.

*I made a try just recently. Went for a bit of a stroll with my companion, and I suffered for it for the next two or three days. Even if everybody gets aches and pains after exercise, it’s more of a problem for me, of course, because I’ve got a lot of spasticity in my legs and when the aches and pains start, the spasticity gets even worse.* (Johnny)

Lack of money was mentioned by some participants as a factor affecting their ability to exercise in that it was costly to use a gym, even for those who had reasonably good economic resources. There was still problems from lack of help and support as well as the problem of the gym’s facilities not being designed for people with an MD. If there was a temperature-controlled pool, it might be quite a long distance away. A further aspect affecting exercise in this population was a fear of falling that *limited outdoor exercise* like walking.

### Strategies based on decisions and attempts

The participants talked about different strategies to reach or maintain a healthy weight. There was great variety in what they *tried to do* or actually *decided to do* to lose weight or maintain their present weight. They spoke of how they tried to eat wisely, tried to exercise as good as they could, tried to eat regular meals and tried to think long-term. They had tried a low-carbohydrate diet and other diets, and they had tried diet pills. One woman described her attempts in this way:

*Well, I’ve tried things you drink. Just imagine what it’s like — you just drink and drink and drink* [laughs] *and never get to chew.... You pretty soon get tired of that. I’ve tried starving myself too. I don’t eat much, but it just doesn’t seem to help.* (Britt)

Some of the participants had firmly decided to reach a healthy weight or to maintain their current healthy weight. These people talked about specific strategies to reach their target. They received professional help from dieticians, and they had *regular mealtimes* and ate moderate portions. Some of the participants *believed in special diets*, such as the 5:2 diet (which means fasting two days weekly) and the LCHF (Low-Carbohydrate–High-Fat) diet. Others had *no faith in special diets*. *Long-term perspective* was described as an important factor with regard to reaching a healthy weight. In addition, the participants spoke of actively choosing healthy food and actively rejecting unhealthy food:

*I always make the necessary adjustments to my life when I’m going to do something, and that goes for my weight too. So I try, I choose — I eat unsweetened muesli, for instance, and I always drink low-fat milk... although full-fat milk’s got a damned better taste.* (Therese)

Some people mentioned that they restricted their food intake because they could not exercise sufficiently. As one participant put it:

*I cut down on what I eat instead. I’ve learnt to do that because I can’t exercise.* (Michael)

Those participants who were able to engage in some kind of *physical activity or exercise* mentioned walking, working out at a gym and doing water aerobics as the most common physical exercise as part of a strategy to reach or maintain a healthy weight. A couple of the participants exercised with friends as a part of their strategy because this approach made it more difficult to skip the activity.

### Internal resources

*Awareness, motivation, attitude* and *general thinking* about the question of weight played a significant role in the participants’ attempts to reach or maintain a healthy weight. The participants had a clear notion of their own ideal weight and were aware that weight gain was to be expected if they used a wheelchair. They were also aware that if they consumed more calories than their body burned off, they would experience an increase in weight. However, it could be difficult to eat less because “food tastes so good”. While some participants refused to count calories, others did so as a way to increase awareness of their energy intake:

*Oh yes, you perfectly automatically keep an eye on what you’re stuffing yourself with all the other days — it just comes naturally. But now I’ve been on holiday, and it was “all inclusive”, which wasn’t good* [laughs]……. *(continues about awareness)*

*Absolutely! You stop and think. “what about that cake... No, we shall not eat that cake today, we’ll skip it today.”* (Eva)

Motivation was perceived by the participants as a need for a specific reason to change. For example, reduction of pain or simply being able to fit into your jeans again. A positive attitude could make a difference. The participants mentioned how they tried to look on the bright side and to see challenges rather than obstacles. Even though it is not always easy to have a positive attitude, you have your life to live.

*I can give up eating, give up drinking Coca-Cola, everything. But what is my life then worth? I would have nothing left.* (Johnny)

Furthermore, the participants had visions of what life would be like if they weighed less: easier movement with less strain on the joints, perhaps also lower blood pressure and lower cholesterol.

### External resources — Experienced and required

The path to a healthy weight was not always straightforward, and the participants needed different forms of support from family, friends and healthcare professionals. Several participants pointed out that it was their own decision to reach or maintain a healthy weight, but they could get support from family and friends (*informal support*) if they wanted it. Support regarding food intake, such as eating the same food as the rest of the family, was emphasized by the participants as the most important form of support. When it came to exercise, however, it was friends who played the major role.

The participants had both negative and positive experiences of *formal support* from contacts with primary health care as well as specialized medical care regarding weight problems. Weight problems had been discussed at meetings with physicians, nurses, physiotherapists and dieticians. But several participants felt that such problems were not really discussed at all in a prevention or treatment perspective. Participants who received treatment for diabetes and/or hypertension who had an established contact with primary health care services said that they had never been offered help for losing weight.

Some participants had been encouraged by the primary healthcare staff to lose weight, but were not offered any proper advice on how to do so. In the participants’ experience, the staff evidently did not see them as being fat enough to be in need of such advice or did not give being overweight a high enough priority. Some participants thought that the health professionals did not have the competence to give appropriate advice about weight problems.

*Then, there was a chat about lifestyle, something like that. But they didn’t seem to get it that you’re disabled, so to speak. They sort of think: “Well, you are just in need of physical activity.” But of course, that’s just the problem! I can’t move the way you want me to. And the dietitian... — she simply didn’t understand, to put it bluntly.* (Elisabeth)

The participants perceived advice from the professionals as contradictory to dietary guidelines, and they felt that the professionals were on high horses with admonishments like “You should do this!” and “You should do that!” In addition, participants sometimes felt that they were not listened to and were even treated insultingly. One participant said that she had tried to have a discussion with the dietician but that it was a one-way discussion:

*I felt she thought I was just babbling and didn’t know what I was talking about.*

*She wasn’t listening to me. I was trying to explain to her that I believe in doing things in a bit of a different way.* (Maria)

The participants’ experiences were not all negative, however, even though such experiences were dominant. Some participants received adequate help for pain rehabilitation and experienced encouragement from their physicians, received good practical advice from dieticians, were treated in a friendly way and were offered follow-up meetings.

*I suppose most of the support came from the hospital. It was a big help. Five, six, maybe seven times I sat there talking, discussing. It gave me an awful lot, both suggestions and encouragement.* (Ingemar)

When it came to *required support*, participants mentioned the importance of a holistic approach in which physicians, nurses, physiotherapists and dieticians were involved:

*I’d like there to be a physician, a dietician and a physiotherapist. Working together, that is. So that they’ve got a grasp of everything at the same time, not just of a bit here and a bit there. So that there is an overall plan for you.* (Elisabeth)

It was pointed out that a holistic approach should include specific knowledge about disability and pain, including things such as correct exercise and nutritional advice from the perspective of managing pain.

*Then there’s the question of food, of what you maybe ought to bear in mind. Perhaps they know what you ought to avoid because it will make the pain worse.* (Kristina)

The criticism of the primary health care centres did reflect the kind of support the participants expected to receive. In particular, the lack of knowledge about physical disabilities was experienced as a disappointment. One man said he wanted specialists:

*But take somebody like me, with a spinal injury. What I regret when it comes to the primary health care centre is that nobody is knowledgeable about my disability.* (Johnny)

## Discussion

In this study, the theme ‘The complex trajectory to a healthy weight’ summarizes the circumstances that govern the attainment and maintenance of a healthy weight. A proper understanding of the complexity is indispensable when it comes to adapting a health promotion intervention for the needs of a person with an MD. Despite difficulties in comparing qualitative studies due to their different questions, similarities were found in a study by Mudge et al. [30]. Even in that study, the participants expressed an added layer of complexity that presented additional factors that needed to be weighed in their choice of living well. The participants in that study talked about multiple factors that influence living well besides healthy food and physical activity. Thus, the complex circumstances need to be illuminated, and the focus in the present discussion will be on pain, the teamwork required for professionals and self-efficacy. Complexity and complex interaction seems to be key terms for when people with an MD discuss their situations both in the present study and in similar studies [29, 30]. Most of the participants in the present study perceived that being overweight or obese increased their pain and exacerbated their MD. This finding is in line with previous literature, which showed that increased daily pain was related to increased BMI [35] as well as an exacerbated MD [23]. The participants also stated that the pain was a barrier to exercise, which was also found among obese African American women with an MD in an article by Rimmer and collages [19]. Similar results were found in an article about physical activity beliefs in which the results showed that one of the behavioural disadvantages with physical activity was increased pain [36]. That study included people with various kinds of MD. Thus, the present study contributes to a deeper knowledge of the described complexity of problems with pain and physical activity related to being overweight and obese. This knowledge indicates there should be future adaption and understanding of health preventions for people with an MD by health professionals. As both pain and further stress on the MD make it more difficult to exercise and since physical inactivity is identified as a risk factor for mortality [18], pain rehabilitation for the purpose of increasing physical activity is necessary for people with an MD.

Even though the findings revealed that pain was a barrier to the performance of physical activity or exercise, physical activity and exercise could be used as a way to reduce pain. A Cochrane review has indicated that physical activity and exercise reduced the severity of pain and improved physical function in patients with chronic pain [37]. Due to the complexity of the combination of pain and physical activity/exercise, the participants in the present study require staff with special skills in pain treatment. Research has shown that multidisciplinary treatment [38] as well as interdisciplinary treatment of chronic pain is more effective than standard medical treatment [39]. However, interdisciplinary teams have shared goals [40], and staff from a broad range of disciplines, such as physicians, registered nurses, physiotherapists, occupational therapists and psychologists, are often included in interdisciplinary teams [41] and should therefore satisfy the participants’ needs. Interdisciplinary teams including dieticians have even been shown to be effective for weight loss [42]. Thus, there is knowledge about both physical activity, pain treatment and interdisciplinary treatment and/or teamwork, and despite these findings, the participants did not receive adequate help from primary health care (PHC). One reason could be that interdisciplinary teams with special skills are not established at the PHC level at which the participants receive medical care, and the best practices of interdisciplinary primary care teams are still being researched [43]. The present study will therefore contribute to a discussion about the need for interdisciplinary work at the PHC level in Sweden to facilitate future interventions through an adapted health prevention program for people with mobility disabilities that is performed by PHC professionals. In Sweden, PHC professionals are responsible for pursuing equality perspectives and health preventions with regard to living habits such as nutrition and physical activity [44]. The lack of preventive work reported in this study indicates that interventions are needed to decrease the number of overweight individuals and associated problems, especially for people with an MD.

The participants in the present study were also dissatisfied with the incompetence of professionals in terms of adapting advice to individuals with an MD. A recently published pilot study concerning the adaptation of a lifestyle intervention for adults with MD showed promising preliminary results with regard both to feasibility and to potential effectiveness for promoting weight loss among people with an MD [45]. However, the intervention included four major adaptations for an MD: telephone meetings, physical activity adapted to the needs of people with an MD, nutrition and health guidelines for wheelchair users and arm-based activity trackers instead of pedometers. Several of these promising findings could be used by health professionals in PHC and should therefore be investigated in such settings.

Several participants talked about what they tried to do when it came to controlling their weight. This “tried” can be interpreted as implying a lack of self-efficacy. Self-efficacy is described in the literature as playing an important part in nutritional behaviour for people either with or without an MD [46–48]. The participants in the present study spoke of their visions, and with a strengthening of self-efficacy such visions could perhaps be turned into reality. One strategy to increase self-efficacy has been shown to be stress management [49]. However, it is therefore important that health professionals be aware of patients with low self-efficacy to support and strengthen their self-efficacy, especially in people with an MD who have a more difficult situation. An interdisciplinary team does often include a psychologist or behaviour therapist [41], and because of the complex situations described in present study, an interdisciplinary team may be the ideal way for people with an MD to reach and maintain a healthy weight.

Because of the complexity of the situation, healthcare professionals need to be well-informed about not only the patient’s weight and MD but also their nutrition, nutrition behaviour, pain and exercise/physical activity. This approach could lead to patients feeling that they are listened to and a reduction in negative symptoms related to being overweight and the MD. As discussed, to address the complex situations among people with an MD, an interdisciplinary team might be one solution. To the best of our knowledge, health professionals working as a team in PHC to reduce being overweight and obese in patients with an MD have not been investigated and are therefore urgently needed.

### Methodological issues

The benefit of the qualitative research method is that it makes it possible to capture the actor’s point of view [32]. However, obesity can be a sensitive topic to discuss, and people can feel guilty. Therefore, people with an MD and normal weight were invited to participate as well so that those who were overweight or obese did not feel that a finger was being pointed at them. At the same time, this approach provides further depth to the exploration of knowledge about people’s own resources and strategies with regard to reaching and maintaining a healthy weight. Thus, choosing participants with diverse weight classes enhances the credibility of the study [50]. Individual interviews were chosen as the data collection method because of the sensitive nature of the topic, and the interviewer’s impression was that the participants felt comfortable talking about their weight.

Participants were selected from the SPHS and had an MD according to admission criteria. There were, however, a few participants with a severe MD in the study group, and only two participants were permanent wheelchair users. This may limit the transferability of the results [50]. Various ways of recruiting participants were discussed. The authors initially considered recruiting study subjects from patient organizations, but this approach might have resulted in a larger proportion of participants with a severe MD compared to the general MD population. Two of the participants who had an MD two years or more have recovered from it. They were included because of the valuable experience they had to offer.

The initial data analysis by the first author was reviewed independently by two researchers (GA and MS), and a discussion of the findings took place in the form of an open dialogue between all authors. Throughout the analytical process, the interviews in their entirety served as a point of reference when a deeper understanding was needed of the meaning units, codes, sub-categories and main categories. Furthermore, the final findings were reviewed by all of the authors. The quotations were used to make the results more credible [51].

## Conclusion

People with an MD in this study experienced a complex situation with pain and exacerbated MD symptoms when an MD and being overweight or obese coexisted. They experienced a lack of support from health professionals, so they tried to reach and maintain a healthy weight by themselves. They requested adequate knowledge about their MD and pain from the health care professionals. We hope that the narratives from the participants uniquely contribute to illuminating the complex situation experienced by individuals with an MD and may facilitate future evaluations of a health prevention program designed for people with an MD combined with being overweight or obese. Evidence-based prevention programs possibly implemented by interdisciplinary PHC teams might become valuable for this group of people with an MD combined with being overweight or obese.

## Abbreviations

BMI: Body mass index
MD: Mobility disability
SPHS: Skåne Public Health Survey
